# Statistician expert witnesses in agreement on relative hazards

**DOI:** 10.1258/cr.2012.011056

**Published:** 2012-03

**Authors:** Sheila M Bird, Jane L Hutton

## Abstract

‘If anticoagulants had been administered sooner, my client would not have died’ was a central claim put to us, as statistician expert witnesses, by a Claimant's and Defendant's lawyers. To assist other litigants, and without identifying the specific case, we set out the study types that contribute to the evidence base, and their limitations. We then explain why it is difficult to adduce evidence about the relative risk of dying from pulmonary embolism within 12 hours of admission to accident and emergency even when it is well accepted that anticoagulation reduces the risk of dying within the next seven days of patients at objectively confirmed risk of pulmonary embolism. No matter how much we may want an answer, or how tragic an individual outcome, we can only work from the available evidence or work to improve the evidence base, which needs to be resourced.

## Introduction

‘If anticoagulants had been administered sooner, my client would not have died’ was a central claim put to us, as statistician expert witnesses, by a Claimant's and Defendant's lawyers.

One of us (JLH) has considerable experience of serving as a statistician expert witness, mainly when life-expectancy for individuals with cerebral palsy is at issue. In cerebral palsy, rigorously analysed, well-collected registry data^1–3^ provide common ground, which helps litigants to achieve an expeditious and equitable resolution.

The other (SMB) has accepted instruction rarely, such as when an anti-doping issue in sport turned on the design of robust surveillance of cattle at slaughterhouses.^4^ In that instance, practical advice on being an expert witness was sought from JLH and also obtained by reading the first part of the Royal Statistical Society's guide.^5^

The case in common was settled; and it was suggested to SMB that lawyers might find it helpful to have an accessible account of the statistical issues therein. By that time, SMB was aware that JLH had also prepared a report.

Without identifying the specific case, we set out why it is particularly difficult to acquire empirical evidence about the risk of death from acute pulmonary embolism (PE) within six or 12 hours of admission to Accident and Emergency (A&E), with or without prescription of coagulation. In part, the difficulty arises from a pharmaceutical success so dramatic that further well-designed, epidemiological research became prohibitively expensive.

## Case history: in brief

Consider a patient who has undergone a hospital procedure which is known to increase the risk of venous thromboembolism (VTE). The patient has been advised to be active at home, rather than sedentary; and, if he experiences symptoms, to return immediately to A&E.

Some days later, the patient experienced shortness of breath, called an ambulance, and was admitted to A&E. Despite his medical history, differential diagnosis did not include (the risk of) pulmonary embolism (PE), nor did an episode of loss of consciousness promote PE above heart failure as presumptive diagnosis. Anticoagulation was not prescribed in the interim while another medical opinion was awaited. The patient collapsed, and cardiac arrest caused death, all within 12 hours of admission to A&E. Autopsy confirmed massive PE.

## Contention

Had anticoagulation been prescribed in A&E prior to the patient's collapse, then – on the balance of probabilities – the patient's death from acute PE within 12 hours of admission to A&E would have been averted. We were separately instructed by the opposing sides to evaluate the evidence for this contention.

## Challenge

The question asked of one of us was: ‘How long is it before injection with heparin prevents death from PE? Subcutaneous injection with low-molecular-weight heparin (LMWH) is absorbed within 30–60 minutes of injection.’ Clinicians, statisticians and lawyers will promptly think of ways in which the question should be refined.

For example, will all deaths from PE be prevented? Should the patient's age and sex be taken into account? Is the disease which the hospital procedure was intended to treat relevant? Are there other aspects of the patient's health or hospital care which are important? These are serious considerations, and natural ones when considering a particular person. However, we show below that it is difficult to answer even the simple question: ‘How long does it take for subcutaneous injection of LMWH to prevent death from PE?’

Almost always, a statistician expert witness will wish to clarify or refine the questions she is asked. The contention and the question above are subtly different. The contention includes assumptions about the decision to prescribe anticoagulation, and the timing of both the decision and the administration of anticoagulant treatment. The question is simpler. If the question cannot be answered, then the further issues of delay in diagnosis and of prescription or administration may be hardly worth pursuing.

## Schematic

Below is a schematic for the progression of venous thromboembolism (VTE) through five stages.

1. Venous thromboembolism (VTE);
2. Symptomatic VTE;
3. Pulmonary embolism (PE);
4. Symptomatic non-massive PE;
5. Symptomatic massive PE.

Not all five stages need be experienced by every patient. Not all patients survive to be admitted to hospital. The patient's PE risk may, or may not, be recognized. Anticoagulation, either before or after confirmed PE diagnosis, may reduce the risk of non-fatal PE recurrence or PE death.

From any stage in the evolution of VTE, a patient may:

- Be admitted to A&E;
- Experience sudden PE death (with or without A&E admission);
- Experience non-PE death (with or without A&E admission).

Non-PE death includes death from cerebral haemorrhage, which can itself arise from the administration of anticoagulants. Morbidities which do not lead to death are also possible in relation to anticoagulation.

## Statistical issue

Highly time-specific hazard rates have to be compared, that is: the risks of death in specified short intervals, such as 2–4 hours after admission or administration. For this comparison, the start time is properly A&E admission. The relevant risk set is patients (with different co-factors) who were admitted to A&E and for whom discharge diagnosis includes PE, and who were (or were not) administered anticoagulation, such as subcutaneous injection of LMWH, at some elapsed time after admission to A&E.

To answer the question, data are required on the time of injection of heparin for a relevant group of patients, and the time and cause of death, or the time when the patient was last known to be alive, which is often the time of hospital discharge.

To address the contention, data are required which record several times for a well-defined group of patients who were admitted to A&E: the start time, which is A&E admission still; the times of diagnosis, prescription and injection of heparin or other anticoagulant; and time to hospital discharge or death. Patients should all have been admitted to A&E, and had a discharge diagnosis which included PE. Additional information for each patient – including age, sex, general health, medical care, and cause of death for those who died – would be necessary.

## Scientific literature

The Appendix (online-only) illustrates the types of evidence available in the scientific literature, together with their strengths and limitations for addressing the contention.

The types of evidence include randomized, dose-ranging studies in young, healthy volunteers. In such studies, the injection time for LMWH, the dose injected, and appropriate blood levels at specified sampling times thereafter are recorded. Blood level data are a surrogate for when LMWH can be expected to begin to reduce the risk of death from PE. Specifically, estimation focuses on how blood levels evolve with time after administration of the randomly assigned dose; and, for a given dose, how variable the time course is between individuals. Translating results from healthy volunteers to at-risk patients with co-morbidities for whom the medication is ultimately intended is at best indirect and so the next study type is a trial in at-risk patients.

A randomized controlled trial (RCT) of a novel intervention in at-risk patients, for whom diagnosis has been objectively confirmed, will occasionally provide such convincing evidence of efficacy that continued randomization to the control group would be unethical. This was the case in the late 1960s for intravenous heparin in PE-diagnosed patients.^6^ Later randomized studies focused on comparing different forms of the intervention (such as intravenous versus subcutaneous injection, or different LMWHs). As the PE-fatality rate within seven days of randomization would be expected to be low, surrogate outcomes – rather than death or PE recurrence – might again be invoked because of the large number of eligible patients required to discern a modest differential impact on an already low death rate.

A second tranche of the scientific literature concerns non-randomized observational studies, which have the advantage that they not only recruit many patients but also reflect the range and reality of clinical practice. As in RCTs, registries may insist on objective confirmation of diagnosis as an eligibility criterion but, unlike RCTs, registries do not have such a natural start time as the time of randomization from which to begin their follow-up of patients, all of whom necessarily survived long enough after hospital admission for their VTE diagnosis to have been both suspected and confirmed objectively.

We now illustrate the analytical consequences which arise from (hypothetical) Registry R's not having recorded the exact times of: i) hospital admission, ii) objectively confirmed VTE diagnosis, and iii) VTE intervention, let alone of iv) fatalities.

To be eligible for Registry R, patients had to have survived the elapsed time (of unknown duration) between i) hospital admission and ii) objectively confirmed VTE diagnosis. The mean elapsed time may have been different according to the intervention which R-registered patients actually received, or according to their VTE progression at hospital admission. In particular, for patients who received LMWH, who were the majority, we do not even know whether, on average: iii) time of administration of LMWH preceded, or followed, ii) objectively confirmed VTE diagnosis. Nor do we know which zero-time [i), ii) or iii)] marked the start of follow-up for R-registered patients who received LMWH.

Table 1 shows a range of assumptions about how many hours, on average, R-registered LMWH-treated patients were actually at risk of PE fatality on Day 0 (hospital admission day) after having received LMWH. If we assume they were at risk for 24 hours, then we obtain the biologically improbable result that PE fatality rate on Day 0 was significantly lower than on Day 1. By assuming an average of 12 hours at risk on Day 0, we would estimate broadly equal hazard rates on Day 0 and Day 1, whereas, if we assume that, post-intervention, R-registered LMWH-treated patients were at risk for an average of six hours only on Day 0, then we'd conclude that the hazard rate per 10,000 patient-days, in that six-hour period, was significantly greater than the time-specific risk on Day 1.

**Table 1:** PE fatalities (with censoring of other-cause deaths) for (hypothetical) R-registered patients whose VTE diagnosis was objectively confirmed

Epoch	Events	Patient-days (pds)	Event rate per 10,000 pds	Poisson 95% CI for event rate per 10,000 pds
Low Molecular Weight Heparin (LMWH): 16202 patients				
Day 0: 24 hrs	27	16202	16.7	11.0 to 24.2
*Day 0: 12 hrs*	*27*	*8101*	*33.3*	*20.0 to 48.5*
Day 0: 8 hrs	27	5400.7	50.0	32.9 to 72.7
Day 0: 6 hrs	27	4050.5	66.7	43.9 to 97.0
Day 1	50	16169.2	30.9	22.9 to 40.8
Day 1 + Day 0: 12 hrs	77	24270.2	31.7	25.0 to 39.7
Day 2 + 3 + 4	105	48174.0	21.8	17.8 to 26.4
Day 5 + 6 + 7	88	46315.8	19.0	15.2 to 23.4
Day 8 + 9 + 10	36	43902.4	8.2	5.7 to 11.4
Day 11 + 12 + 13	12	43773.5	2.7	1.4 to 4.8

Table 1 shows a further reduction in PE fatality rate after Day 7 for the Registry R's LMWH-treated VTE-confirmed patients. Of course, patients' PE fatality risk may be decreasing over time for reasons of differential frailty that are unconnected with the pharmaceutical intervention they received.

In short, analysis of time-specific outcomes for R-registered patients can only be conjectural because we do not know from which zero-time patients' follow-up has been measured. In particular, inference about the mean duration of any LMWH refractory period (i.e. before LMWH-associated PE fatality hazard reduction comes into play) is conjectural – with or without allowance for VTE progression at admission and for patients' age, sex and co-morbidity.

## Discussion

### Other study designs

The available studies do not contain sufficiently detailed information to answer the question of how long it takes ‘before subcutaneous injection of LMWH reduces the risk of death from PE'. The analysis of Registry R's data, see also Laporte et al.,^7^ could, of course, have been improved by detailed recording of times i) to iv). However, the case at issue did not fit the eligibility criteria for Registry R anyway – because the patient's death preceded objective confirmation of VTE diagnosis, which was at autopsy.

The best study design is one which marries the record-linkage credentials of the final study^8^ in the Appendix with abstraction of specific times (if indeed recorded) from both A&E and other hospital notes; and does so for tens of thousands of patients. Many patients have to be included as fewer than four in 100 patients admitted to A&E for whom discharge diagnosis includes PE are expected to die within seven days of admission to A&E; and LMWH treatment is very effective in patients with objectively confirmed VTE, of whom fewer than two in 100 are expected to die within seven days from PE. Times of interest would include biomarker levels measured at protocol pre-specified times after the administration of anticoagulation but would also have to include estimation of the time interval in which PE occurred or re-occurred.

In a busy A&E department, the accurate recording of times (to the minute or even hour) has to compete with other activities, particularly in the case of seriously ill patients who may need rapid administration of oxygen or fluids. At the time of a death, concern for relatives may well take precedence over making notes. If notes have to be completed later, recall of times is unlikely to be accurate to much less than fifteen minutes: even that may be too optimistic. The alternative of having additional staff, with a clerical role^9^ of noting exact times, would be intrusive for patients, families and healthcare teams.

Such a study would also be prohibitively expensive. Employing clerical staff to record precise times, and the taking of additional, precisely timed biomarker measurements, would also be for research purposes rather than routinely required. Hence, the study would require both research ethical approval and informed patient consent. There is a good chance that patient or proxy consent would not be given by a substantial proportion of patients, even with substantial expenditure on gaining consent. For example, consent was given for no more than half of all patients eligible to be on a Canadian Stroke Register, and there were major differences between those enrolled and those who refused.^10,11^

### Costing an accurate record linkage and biomarker study if the day-specific hazard is 1 to 5 deaths per 1000 patients

Study size would need to be sufficient at the lower day hazard rate of one death per 1000 patients. Adequate estimation of hazard rates for each 24-hour period for a specific subgroup requires us to observe around 20 deaths for that subgroup on any specific day. Taking demography and co-morbidities into account could readily give rise to 10 subgroups, and hence the need to document 200,000 patients who were admitted to A&E and whose discharge diagnosis included PE. Allowing £75 for informed consent, biomarker measurements and accurate recording would bring study costs on 200,000 patients to around £15 million.

We can only conjecture at how much the public purse has expended in the past five years on claims of clinical negligence in respect of PE fatalities (see http://www.nhsla.com/home.htm). However, if there were 20 such cases per annum at an average cost of £750,000 each, then it would take, for example, a 20% reduction in litigation costs over the next five years to fund the very large epidemiological study that might be needed to discover how the question should be answered and how clinical guidance^12,13^ operates into clinical practice.

### Reflections

Many questions which are reasonable to ask – how quickly pharmaceutical drugs take effect, what adverse effects arise from those drugs, whether a baby has been intentionally injured by a guardian – are very difficult to answer reliably. For common acute conditions, results might accumulate quite quickly. For chronic diseases, such as diabetes or epilepsy, where adverse effects might develop only after longer term use of a drug, there are no rapid ways of discovering deleterious effects.

If the incidence of death or an adverse effect is common, then relatively few patients need be studied. The NHS's definition of ‘common’ is ‘one in 10 to one in 100’. Reliably to detect a difference between an adverse event rate of one in 10 per month versus one in 20 per month would require recruiting 870 patients and observing them accurately for a month (5% significance, 80% power). To be 95% to 99% confident of ruling out an adverse event rate of one in 1000 (or one in 10,000), some 3000 to 5000 (or 30,000 to 50,000) patients would have to be studied without observing a single event.^4^

There might not be as many patients as are needed to answer a question such as: ‘does controlling type 1 diabetes by insulin pump rather than by injections lead to healthier newborns?' This clearly needs a study of diabetic women who are planning to have children, or have had children. As there are only about 280,000 people in the UK of both sexes and all ages who have type 1 diabetes, the number of UK women who might be included in such a study is not large.

A response to the difficulty of obtaining good-quality scientific evidence on risks might be to seek clinical opinion. A clinical opinion can only summarize the scientific rationale (as currently known), personal experience, and published research. Personal experience of rare events is logically limited.

In assessing risks, and answering questions, prudence is recommended. No matter how much we may want an answer, or how tragic the individual outcome, we can only work from the available evidence or on improving the evidence base cost-efficiently.

**Declarations of interest:** Authors were statistician expert witnesses who were engaged by opposite sides, in the same case.

**Contributions:** SMB is guarantor: both authors contributed to the planning, content and writing and editing of the manuscript.

**Funding:** SMB is funded by the Medical Research Council (programme: MC_US_A030_0007/010).
